# Human Papillomavirus-Associated Vulvar Intraepithelial Neoplasia in an Immunocompromised Patient—When Excisional Treatment Is Not Possible

**DOI:** 10.3390/jpm16100504

**Published:** 2026-09-29

**Authors:** Joana N. Figueiredo, Lúcia Correia, Margarida Bernardino, Patrícia Pinto, Joana Ferreira, Mariana Ormonde

**Affiliations:** 1 Serviço de Ginecologia e Obstetrícia, Unidade de Caldas da Rainha, ULS do Oeste, 2500-176 Caldas da Rainha, Portugal; 2 Serviço de Ginecologia Oncológica, Instituto Português de Oncologia de Lisboa-Dr. Francisco Gentil, 1099-023 Lisboa, Portugal; 3 Serviço de Anatomia Patológica, Instituto Português de Oncologia de Lisboa-Dr. Francisco Gentil, 1099-023 Lisboa, Portugal

**Keywords:** human papillomavirus, vulva, HIV, acquired immunodeficiency syndrome, imiquimod

## Abstract

Human papillomavirus-associated vulvar intraepithelial neoplasia (HPVa VIN) is a premalignant condition. Its main risk factors include human papillomavirus (HPV) infection, smoking, and immunodeficiency. Excisional treatment is preferred, but if contraindicated, topical imiquimod may be considered. The prognosis appears to be similar after these two treatments. Still, there are few studies in women living with human immunodeficiency virus (HIV) to draw a conclusion in this group. We present the case of a woman living with HIV and meeting diagnostic criteria for acquired immunodeficiency syndrome (AIDS), with extensive, multifocal, and recurrent HPVa VIN. Due to anesthetic contraindications, we opted for topical imiquimod treatment, with good results, achieving a complete clinical response after 18 weeks of treatment. Two months later, a clinically suspected recurrence was detected, and imiquimod was reinitiated. Complete clinical resolution was again achieved after 20 weeks of treatment, and the patient started clinical surveillance every 3 months.

## 1. Introduction

Human papillomavirus-associated vulvar intraepithelial neoplasia (HPVa VIN), previously known as high-grade squamous intraepithelial lesion of the vulva, is a premalignant condition strongly associated with human papillomavirus (HPV) infection [1]. It occurs more frequently in premenopausal women, with a mean age at diagnosis of approximately 46 years [1]. The reported incidence ranges from 2.5 to 8.8 per 100,000 women per year and appears to be increasing worldwide [2].

Risk factors for HPVa VIN include HPV infection, particularly high-risk subtypes like HPV 16 (more than 70% of cases) and HPV 33 (approximately 6%), cigarette smoking, and immunodepression, particularly in patients with human immunodeficiency virus (HIV) infection [1,2]. Women living with HIV tend to develop the disease at a younger age and more often present with multifocal and multicentric lesions, as well as higher rates of recurrence and progression, with shorter disease-free intervals [2,3,4,5]. In these patients, the incidence and severity of vulvar premalignant and malignant disease appear to correlate with worsened immunodepression [3,4].

Clinical symptoms and presentation of HPVa VIN are similar regardless of HIV status and may include pruritus, pain, burning, ulceration, or bleeding, although some patients remain asymptomatic [4]. Diagnosis is based on histologic confirmation. Biopsies are essential to exclude invasive disease before treatment [1,5].

Treatment of HPVa VIN is recommended due to an estimated 10% risk of progression to vulvar carcinoma; however, the optimal management remains uncertain [1,2]. Available treatment options include surgical excision, ablative techniques, and topical therapy with imiquimod [1,2]. Surgical excision has traditionally been considered the standard treatment. However, it may result in significant anatomical distortion and morbidity, particularly in cases of multifocal, recurrent, or anatomically extensive disease involving the clitoris, urethra, anus, or vaginal introitus.

Topical treatment with imiquimod may be considered a first-line option in selected cases to preserve normal vulvar anatomy and function, provided invasive disease has been histologically excluded [1,2,6]. Imiquimod is a topical immune response modifier that acts as a Toll-like receptor 7 agonist, activating immune cells and stimulating the secretion of pro-inflammatory cytokines. It alters the local immune response in favor of clearing persistent HPV infection [1,3,6]. A typical regimen consists of application three times per week for at least 16 weeks [1,2]. Limitations to its use include the duration of treatment and the absence of histological specimens. Reported outcomes of imiquimod in the general population appear comparable to those of surgical approaches, although the evidence remains limited. Complete response rates with imiquimod range from 50% to 81% (versus 65% to 91% for surgical treatment), with recurrence rates of approximately 11% to 40% (versus 22% to 60% for surgical excision) [1,3,6,7,8].

In patients living with HIV, higher rates of recurrence and progression to cancer have led to surgical excision being commonly recommended as first-line treatment. Nevertheless, in cases where surgery may result in significant morbidity, topical therapy may be considered [4]. There is little data about the treatment of HPVa VIN in women living with HIV, particularly in those with diagnostic criteria for acquired immunodeficiency syndrome (AIDS). Furthermore, there are no recent published cases about topical treatment in this group of patients. Hence, evidence regarding the management of HPVa VIN in women living with HIV, particularly with imiquimod, remains scarce [4,5,9].

We present the case of a woman living with HIV and meeting diagnostic criteria for AIDS with extensive, multifocal, and recurrent HPVa VIN treated with topical imiquimod due to anesthetic contraindications, which precluded surgical management.

## 2. Case Report

We present a case of a 41-year-old woman, a former smoker, with a history of multifactorial pancytopenia, severe pulmonary hypertension, and HIV infection with AIDS criteria since 2003, namely CD4 cell count < 200/uL, pulmonary tuberculosis and recurrent bacterial pneumonia. In 2021 she underwent surgical excision of a vulvar lesion at a secondary oncology center, with a histological diagnosis of HPVa VIN. Cervical HPV testing was positive for high-risk HPV 66.

In 2023, she was referred to our institution, a tertiary oncology center, due to worsening of vulvar lesions associated with intense pain. At this time, cervical HPV testing was negative for high-risk HPV. She was on antiretroviral therapy with emtricitabine + tenofovir + dolutegravir, but with poor adherence. Laboratory evaluation revealed a CD4 cell count of 123/uL and an undetectable HIV viral load (<50 copies/mL). On physical examination, an irregular, elevated erythematous lesion measuring 5 cm × 7 cm was observed involving the clitoris and the right labia majora. This lesion was located approximately 1 cm from the urethral meatus. Additionally, a 2 cm × 2 cm whitish plaque was identified on the left side, involving the left labia minora (Figure 1A,B).

Two incisional biopsies were performed, one involving the lesion on the right labia majora and the other involving the lesion on the left labia minora. Histology revealed HPVa VIN, excluding malignancy (Figure 2A,B). Vulvar HPV testing was not performed.

Although excisional treatment was initially proposed due to risk factors for progression to invasive disease, it was deemed unfeasible. The poorly controlled comorbidities, mainly the long-lasting pancytopenia with a platelet count of 5.6 × 10^10^ /L and the severe pulmonary hypertension associated with respiratory failure requiring permanent oxygen therapy, were considered anesthetic contraindications for general anesthesia and locoregional anesthesia. Excisional treatment under local anesthesia was considered inadequate given the extent of the lesions. A trial of CO_2_ LASER vaporization under local anesthesia was then suggested, but the patient refused it.

Therefore, topical treatment with imiquimod was proposed and accepted by the patient, after explanation of the risks, benefits, and uncertainties of its efficacy. Imiquimod cream 5% was initiated and applied three times weekly for a planned duration of 12–20 weeks. A trained nurse performed all applications in a hospital setting to ensure compliance. A thin layer was applied to the lesions and rubbed in until the cream disappeared. The patient was instructed to avoid bathing for the next 6 to 10 h. Adherence to treatment was complete, and the patient reported no significant adverse effects. Simultaneously, the importance of strict adherence to antiretroviral therapy was reinforced. The patient was monitored monthly, with progressive regression of lesions (Figure 3A,B), and achieved a complete clinical response after 18 weeks, at which point treatment was discontinued (Figure 4). Given the risk of disease recurrence, the patient began follow-up with a clinical examination every 3 months.

One month after imiquimod was suspended, the patient was hospitalized for severe respiratory infection complicated by pulmonary thromboembolism, resulting in worsening immunodepression. Laboratory evaluation revealed a CD4 cell count of 65/uL and an undetectable HIV viral load (<50 copies/mL). At that time, the patient had suspended antiretroviral therapy emtricitabine + tenofovir + dolutegravir without medical indication.

Four months after the first complete clinical response, clinical evaluation revealed a 1 cm × 1 cm whitish lesion between the left labia minora and majora (Figure 5). This lesion was not biopsied because the patient had a platelet count of 6.6 × 10^10^/L, but its appearance was very suggestive, and it was assumed to be a clinically suspected recurrence.

Topical imiquimod cream 5% was reinitiated three times weekly, one under nurse supervision and two at home, and following the same application instructions. Once again, the patient completed all applications and reported no significant adverse effects. The same antiretroviral therapy was resumed. After 20 weeks of treatment, complete clinical resolution was again achieved (Figure 6). Laboratory evaluation revealed a CD4 cell count of 201/uL and an undetectable HIV viral load (<50 copies/mL). The patient restarted follow-up with clinical examination every 3 months for at least a year. Figure 7 presents a summary timeline of the clinical case described.

## 3. Discussion

Women living with HIV have a higher risk of developing multifocal and recurrent HPVa VIN and experience higher rates of progression than the general population, likely because HIV impairs HPV clearance and promotes viral persistence [3]. Our patient presented several of these features, including extensive, multifocal, and recurrent disease. Furthermore, recurrence occurred after interruption of antiretroviral therapy and worsening immunodepression, supporting previous observations that the clinical course of HPV-related disease is closely associated with immune status [3,4].

Another noteworthy finding was the negative cervical high-risk HPV test despite the presence of extensive HPVa VIN. Vulvar high-risk HPV testing was not possible to perform; so, we cannot confirm which HPV was associated with the HPVa VIN. Although HPV infection of the cervix and vulva is closely related, these sites may follow different natural histories. In our patient, cervical high-risk HPV (HPV 66), detected two years earlier, was no longer identified at the time of diagnosis of recurrent HPVa VIN. Although the underlying mechanism remains unclear, this finding suggests that viral clearance may differ across anatomical sites, particularly in immunocompromised patients.

Although surgical excision remains the preferred treatment for HPVa VIN, previous studies have demonstrated that topical imiquimod is an effective alternative in selected patients, especially when surgery is contraindicated or would result in significant functional or anatomical morbidity [6,7]. In our patient, severe comorbidities precluded surgical treatment, making topical imiquimod a feasible therapeutic option. Another option was a trial of CO_2_ LASER vaporization, but the patient refused it.

One disadvantage of imiquimod is the lack of histological assessment of treatment response; so, clinical remission cannot establish histological clearance. Consequently, this can make it difficult to distinguish between persistent disease and early recurrence. Nevertheless, continued clinical surveillance is essential, and if a suspicious lesion is identified during follow-up, a biopsy should be performed.

Despite the prolonged treatment course, our patient achieved complete clinical remission with imiquimod first after 18 weeks of treatment and then after 20 weeks of treatment post-suspected recurrence. This favorable outcome is consistent with previously reported response rates for imiquimod in HPVa VIN [1,3,6,7,8]. Unfortunately, these studies do not include patients living with HIV or other immunocompromised patients, or include only a small number of such patients. Consequently, evidence regarding the use of imiquimod in women living with HIV, particularly those meeting diagnostic criteria for AIDS, remains scarce, and surgical treatment is generally favored because of the higher risk of recurrence and progression [4,6]. Fernández-Montolí et al. observed that patients with HIV showed more partial responses and HPV positivity after imiquimod than patients without HIV, hence a higher risk of recurrence after topical treatment and a greater need for additional treatments [3].

Nevertheless, our patient achieved complete clinical remission after both the initial treatment and retreatment following recurrence associated with worsening immunodepression. Although conclusions cannot be drawn from a single case, this experience suggests that topical imiquimod may represent an effective therapeutic option in carefully selected patients living with HIV when surgery is not feasible, provided that invasive disease has been excluded and close follow-up is ensured.

This case highlights the importance of an individualized treatment strategy and illustrates how treatment selection can be tailored to the individual patient’s clinical characteristics.

## 4. Conclusions

This case suggests that topical imiquimod may represent an effective treatment option for HPVa VIN in carefully selected patients living with HIV when surgical management is not feasible. Careful patient selection, strict follow-up, and exclusion of invasive disease are essential. Further studies are needed to better define optimal management strategies in this high-risk population.

## Figures and Tables

**Figure 1:**
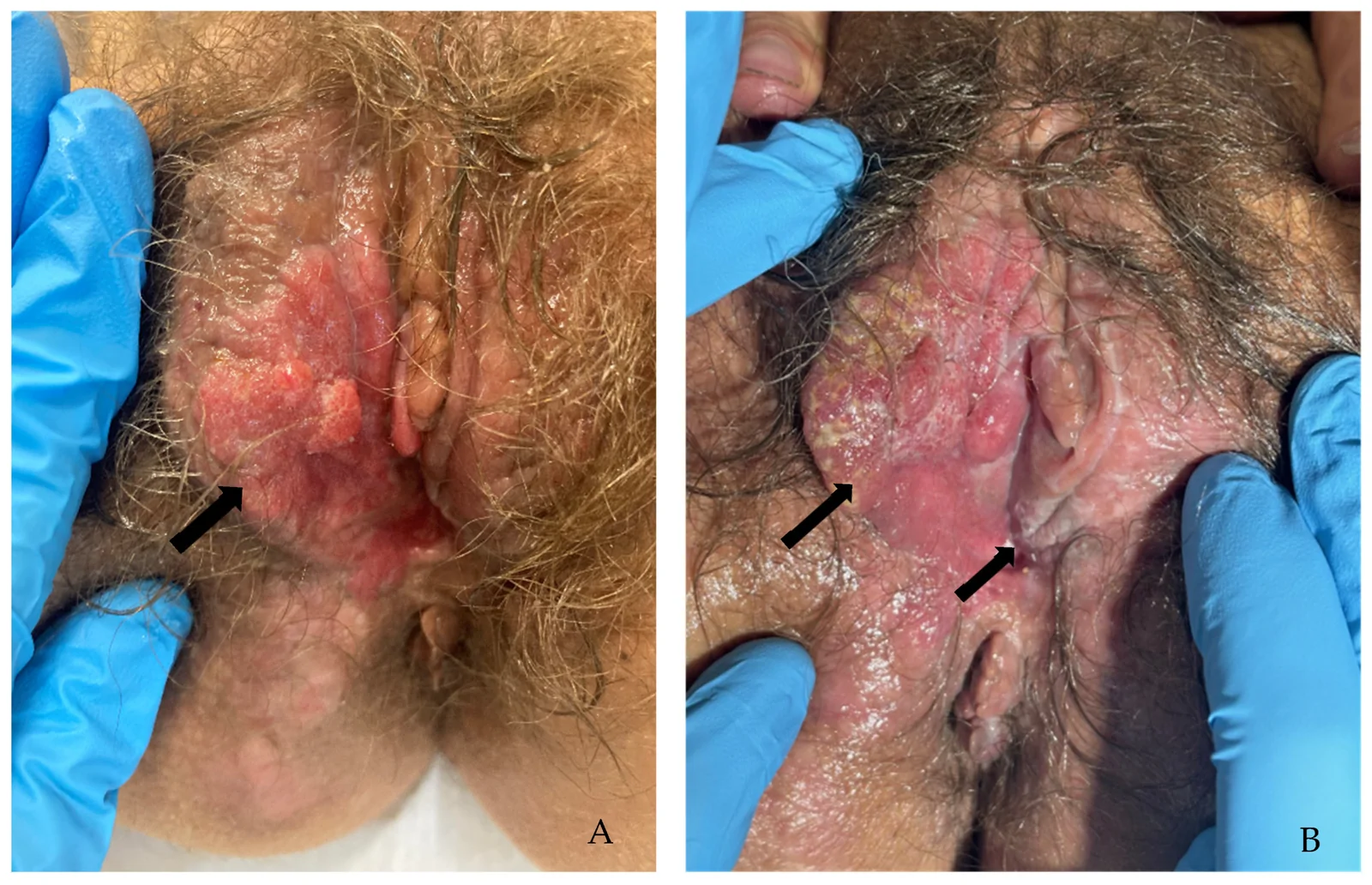
Vulvar lesions, indicated by arrows, before treatment started. (**A**) Erythematous lesion on the right labia majora; (**B**) Erythematous lesion on the right labia majora and a whitish plaque on the left labia minora. The arrows indicate vulvar lesions before treatment started.

**Figure 2:**
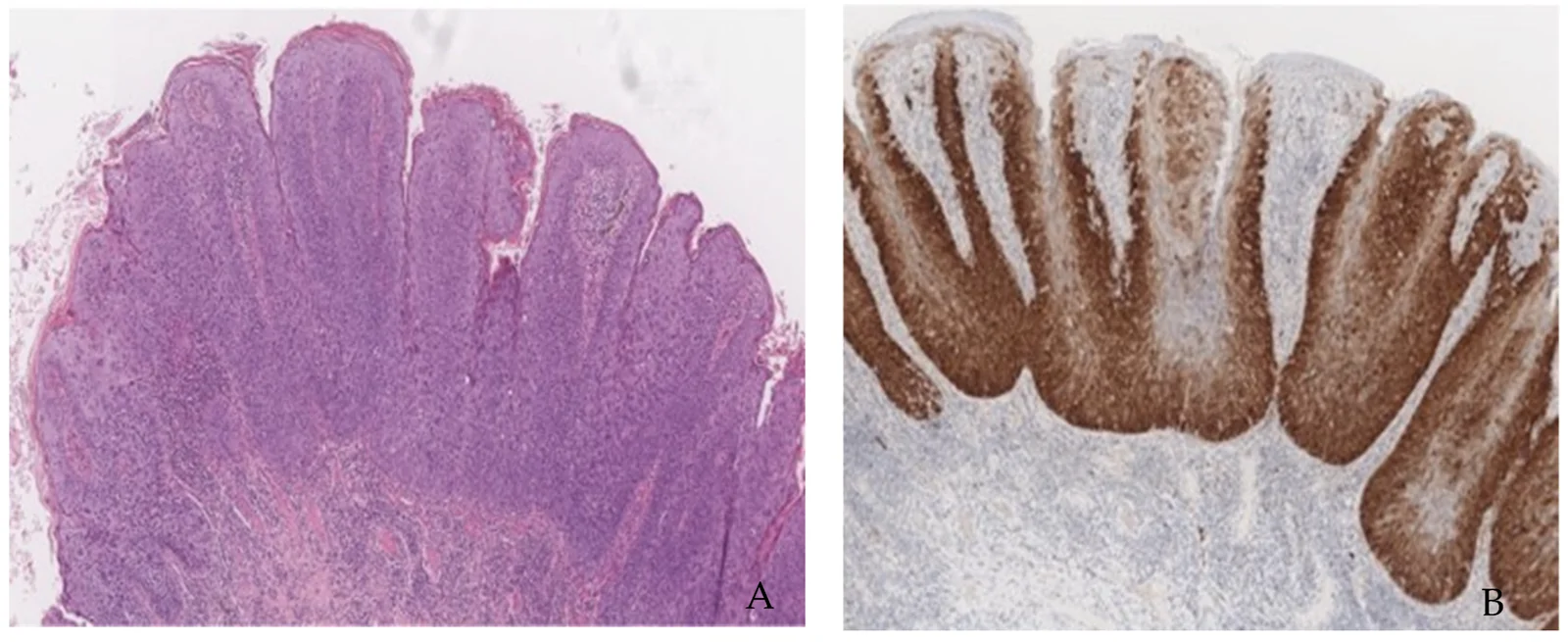
Biopsy with a fragment of mucosa covered by squamous epithelium showing a focus of severe dysplasia. (**A**) Hematoxylin–Eosin staining and (**B**) block positive p16.

**Figure 3:**
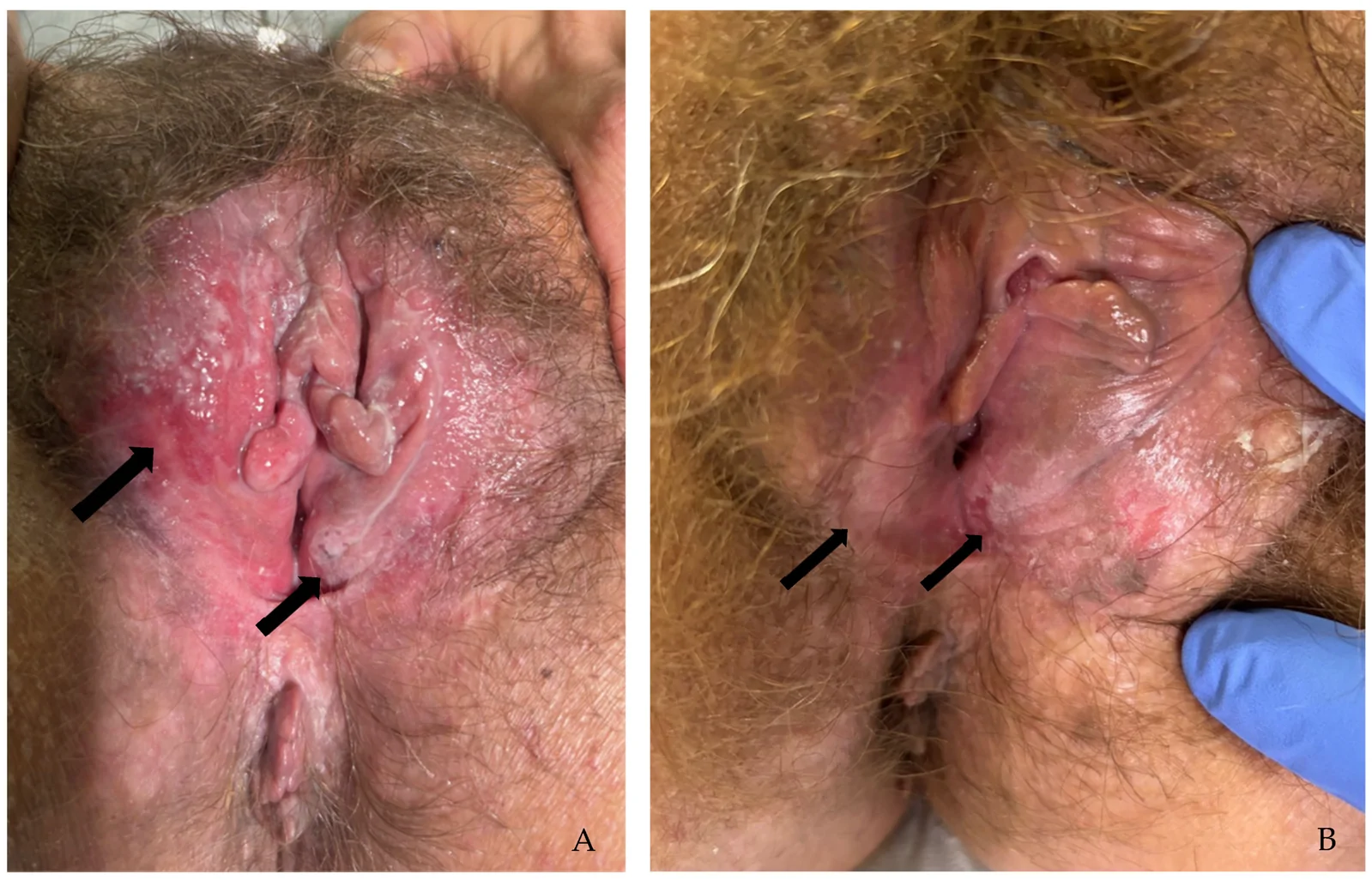
Progressive regression of vulvar lesions during treatment with imiquimod. The arrows indicate the two vulvar lesions initially described in the right labia majora and the left labia minora. (**A**) Vulvar lesions at the 8th week of treatment. (**B**) Vulvar lesions at the 12th week of treatment.

**Figure 4:**
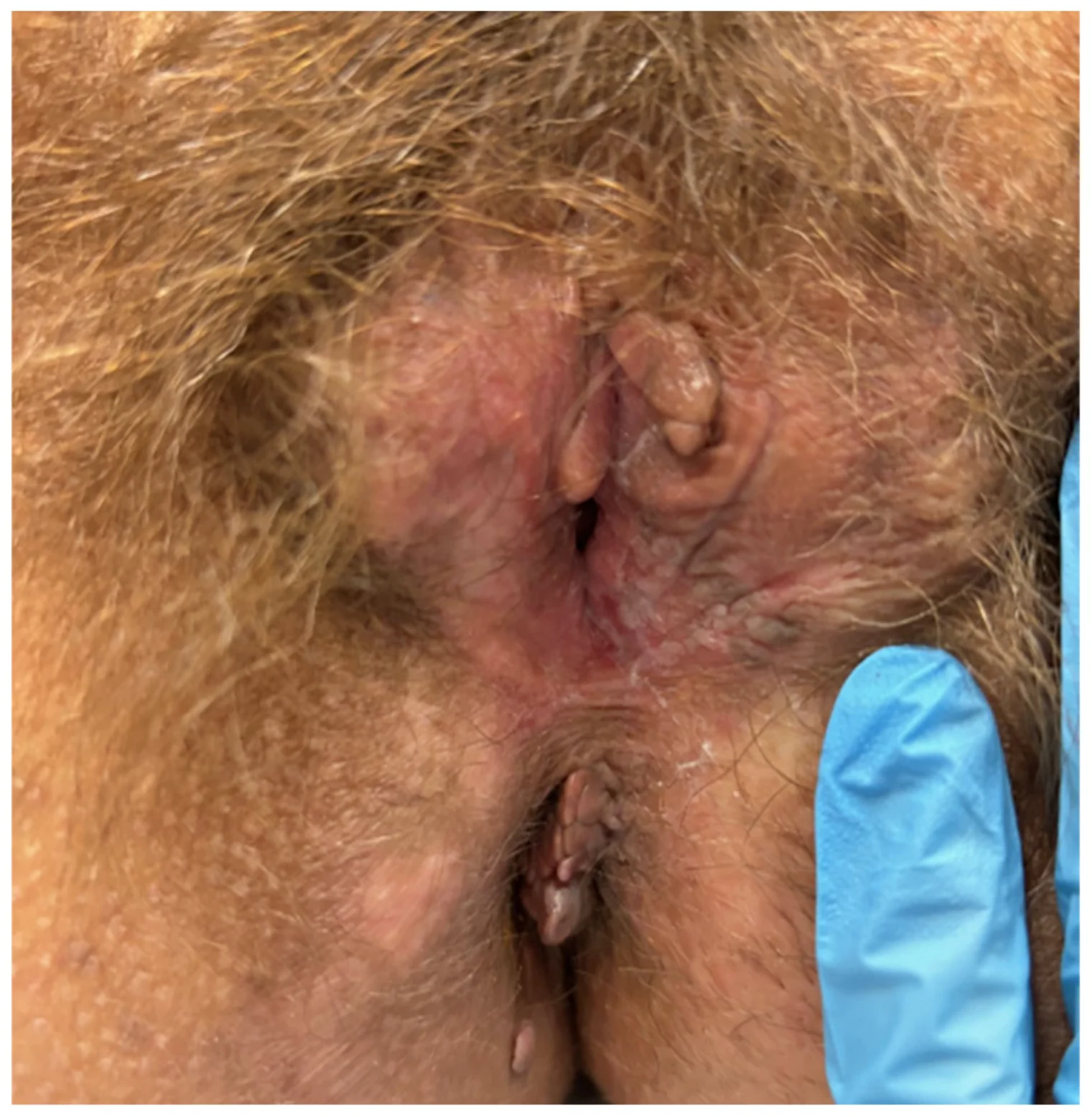
Vulvar lesions at the 18th week of treatment.

**Figure 5:**
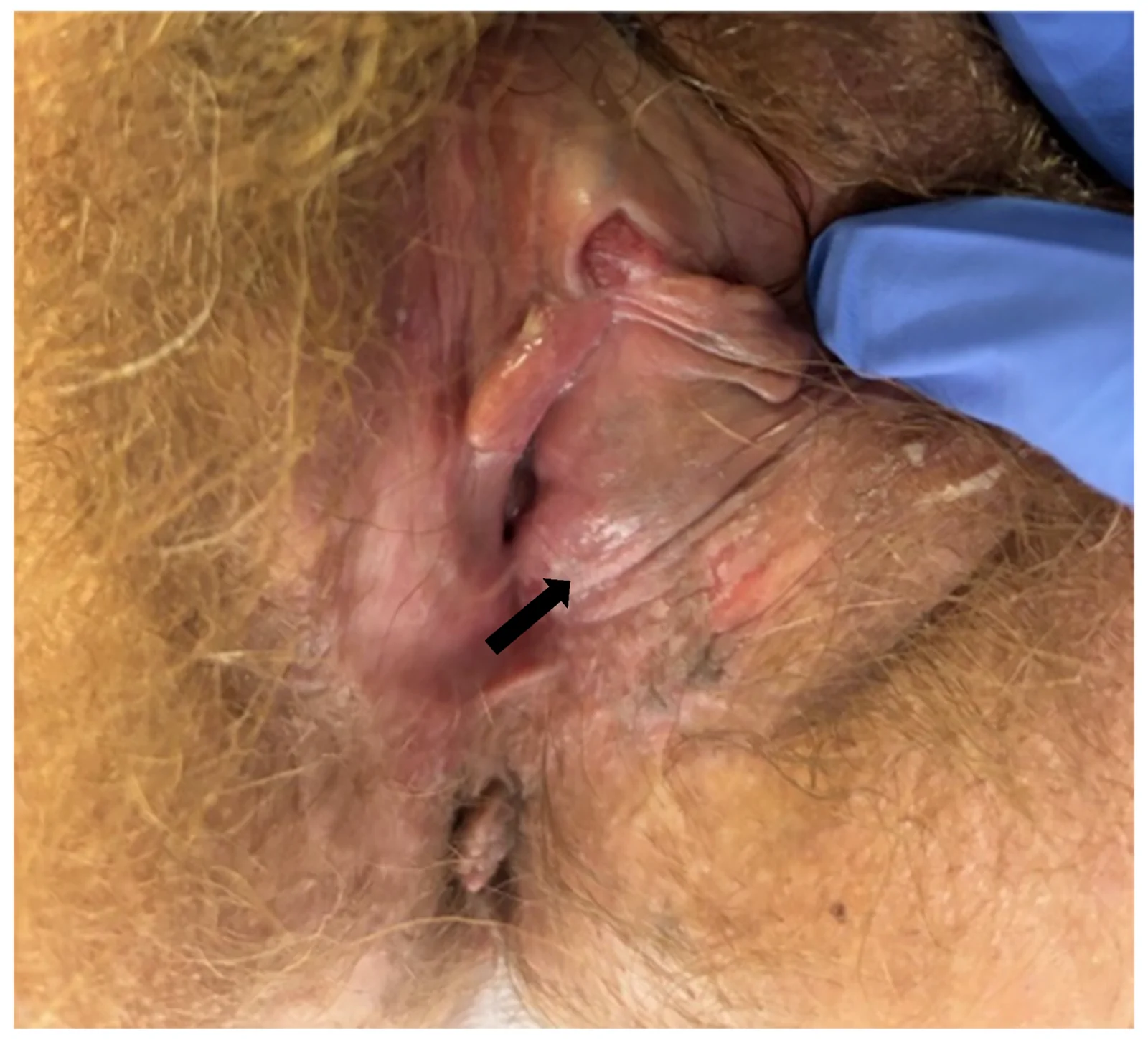
Vulvar lesion recurrence indicated by the arrow.

**Figure 6:**
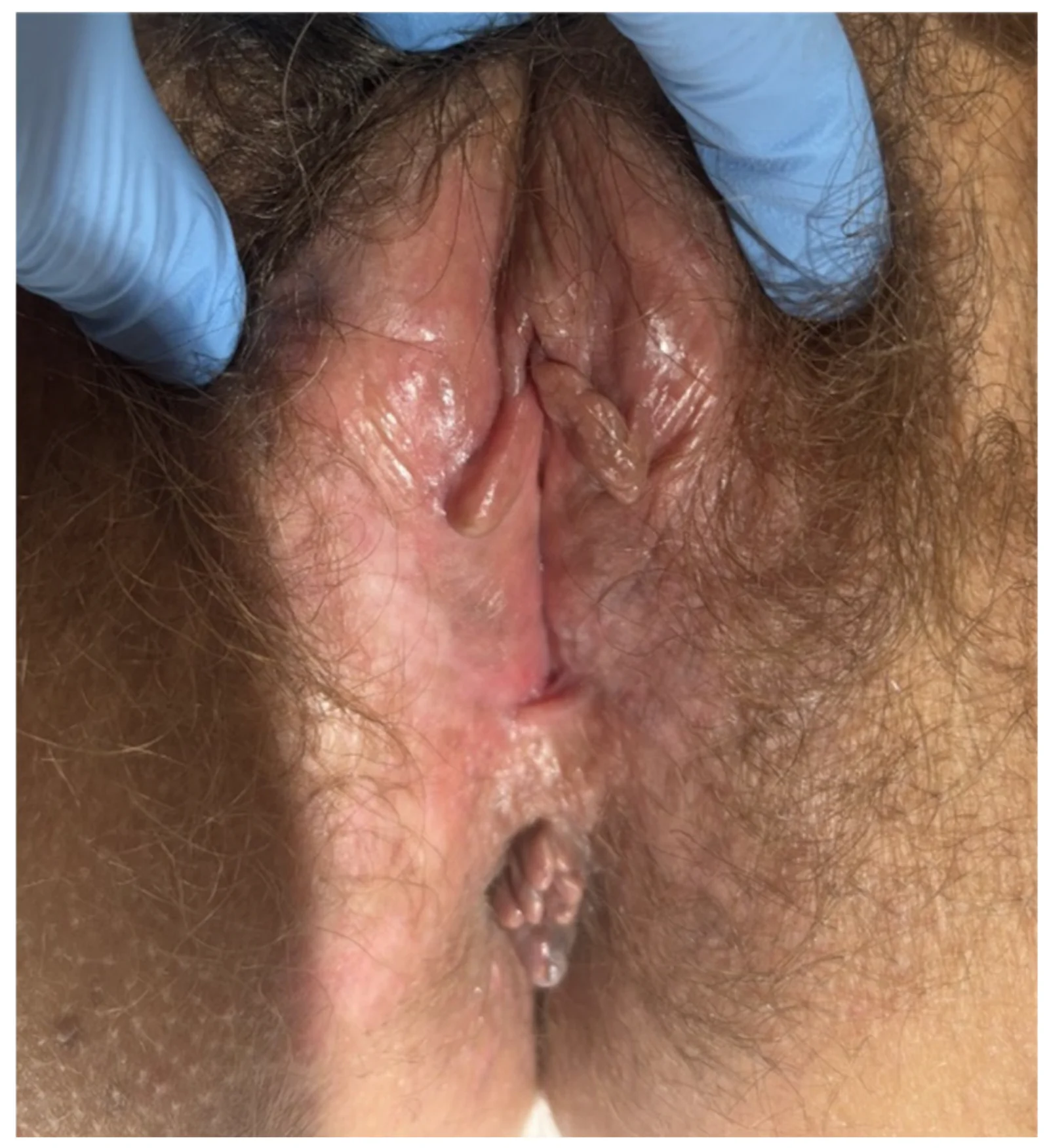
Vulva after 20 weeks of treatment.

**Figure 7:**
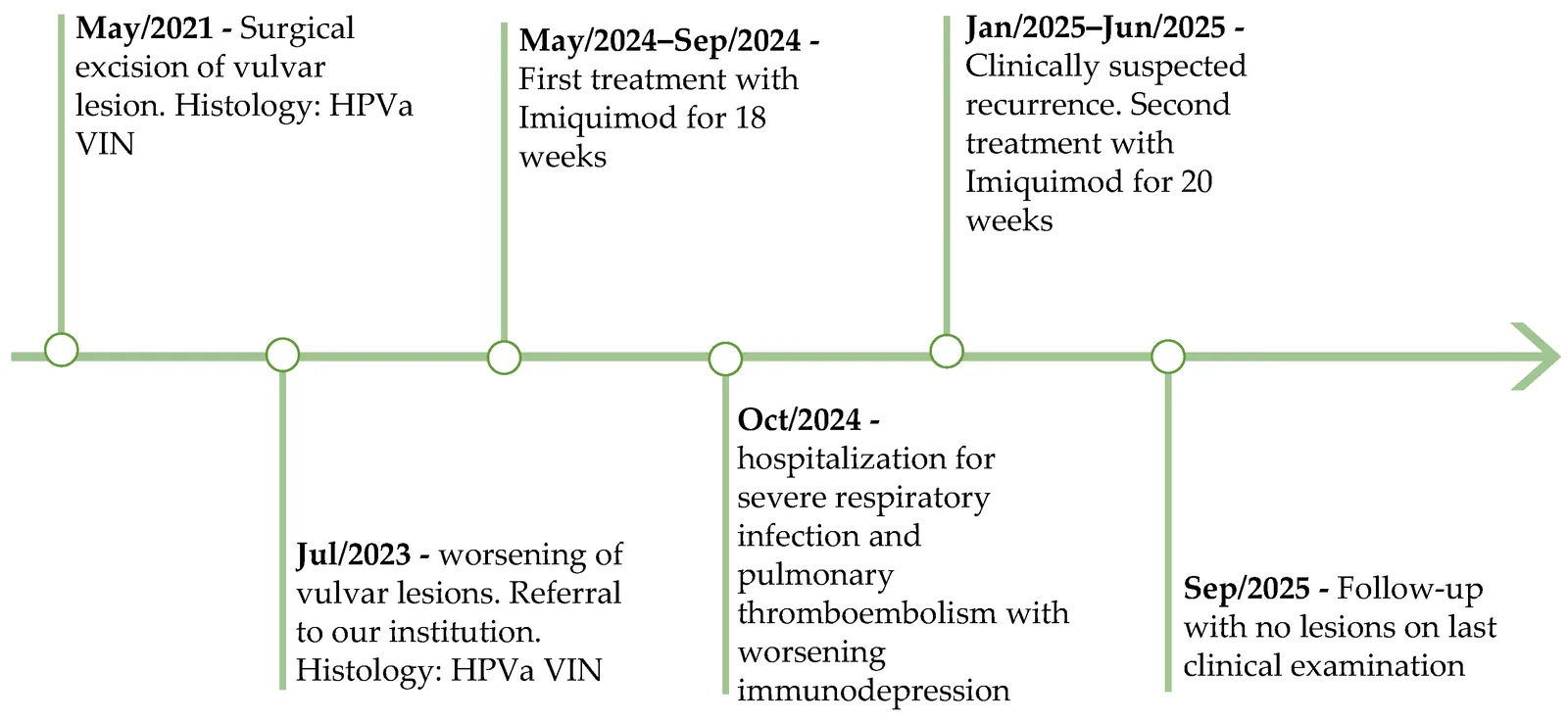
Clinical case timeline.

## Data Availability

The original contributions presented in this study are included in the article. Further inquiries can be directed to the corresponding author.

## Abbreviations

The following abbreviations are used in this manuscript:

HPVa VIN	Human papillomavirus-associated vulvar intraepithelial neoplasia
HIV	Human immunodeficiency virus
HPV	Human papillomavirus
AIDS	Acquired immunodeficiency syndrome
